# Superconductivity in Ta_3_Pd_3_Te_14_ with quasi-one-dimensional PdTe_2_ chains

**DOI:** 10.1038/srep21628

**Published:** 2016-02-15

**Authors:** Wen-He Jiao, Lan-Po He, Yi Liu, Xiao-Feng Xu, Yu-Ke Li, Chu-Hang Zhang, Nan Zhou, Zhu-An Xu, Shi-Yan Li, Guang-Han Cao

**Affiliations:** 1 Department of Physics, Zhejiang University of Science and Technology, Hangzhou 310023, China; 2 State Key Laboratory of Surface Physics, Department of Physics, and Laboratory of Advanced Materials, Fudan University, Shanghai 200433, China; 3 Department of Physics, Zhejiang University, Hangzhou 310027, China; 4 Department of Physics, Hangzhou Normal University, Hangzhou 310036, China; 5 Collaborative Innovation Centre of Advanced Microstructures, Nanjing 210093, China

## Abstract

We report bulk superconductivity at 1.0 K in a low-dimensional ternary telluride Ta_3_Pd_3_Te_14_ containing edge-sharing PdTe_2_ chains along crystallographic *b* axis, similar to the recently discovered superconductor Ta_4_Pd_3_Te_16_. The electronic heat capacity data show an obvious anomaly at the transition temperature, which indicates bulk superconductivity. The specific-heat jump is Δ*C*/(*γ*_*n*_*T*_*c*_) ≈ 1.35, suggesting a weak coupling scenario. By measuring the low-temperature thermal conductivity, we conclude that Ta_3_Pd_3_Te_14_ is very likely a dirty *s*-wave superconductor. The emergence of superconductivity in Ta_3_Pd_3_Te_14_ with a lower *T*_*c*_, compared to that of Ta_4_Pd_3_Te_16_, may be attributed to the lower density of states.

Superconductivity (SC) in low-dimensional systems attracts sustained attention in SC community. The discovery of first layered cuprate superconductor (La, Ba)_2_CuO_4_^1^, has set off a wave of exploring high-*T*_c_ superconductors. Since after, a number of new superconductors with low dimensional structures, such as quasi-two-dimensional (Q2D) strontium ruthenate^2^, ferroarsenides^3^, bismuth oxysulfides^4^, quasi-one-dimensional (Q1D) transition-metal chalcogenides^5^, ternary tellurides^6^,^7^, and newly discovered chromium-based compounds^8^, were reported to display the features of novel SC. The spin (charge) fluctuations^9^,^10^, strong electron-electron correlations^11^, or metal-insulator boundaries^12^ among low-dimensional systems constitute the newly strategic prerequisites to explore high-*T*_c_ superconductors. Generally, the presence of some transition metal elements among them, which bear strong electron correlations, are believed to play a significant role in producing the exotic pairing glue.

Owing to the inherent nature of transition metal chalcogenides^13^, the low-dimensional structures and rich physical properties, e.g., density-wave instability^14^, thermoelectricity^15^, and SC^5^, are prevalent among them. The tellurides, as compared with sulfides or selenides, are quite special in terms of its structures and properties because of the diffuse nature of the tellurium orbitals, and thus far rarely studied. Recently, we reported the observation of SC with *T*_*c*_ = 4.6 K in a ternary telluride Ta_4_Pd_3_Te_16_ with Q1D PdTe_2_ chains^6^. The detailed studies of its pairing symmetry were followed in applying the techniques of scanning tunneling microscopy^16^, low-temperature heat capacity^17^ and thermal conductivity^18^. The results indicate an anisotropic gap structure with the possible presence of nodes, although electronic structure calculations show the contributions of Pd 4*d* electrons to the density of states at Fermi level are pretty small^19^.

From a crystal-structure viewpoint, Ta_4_Pd_3_Te_16_ also belongs to a layered compound resulting from the condensation of Pd-based octahedral chains, Ta-based bicapped trigonal prismatic chains, and Ta-based *double* octahedral chains^6^,^20^. If the Ta-based *double* octahedral chains are replaced by Ta-based *single* octahedral chains, the condensation of the three different types of chains would form the atomic layer of a new compound Ta_3_Pd_3_Te_14_, which was firstly synthesized by Liimatta and Ibers in 1989^21^. The major difference between them in structure is well reflected from Fig. 1(f), which shows the projection view of one atomic layer of Ta_3_Pd_3_Te_14_ and Ta_4_Pd_3_Te_16_ along the *b* axis. The structural details of Ta_3_Pd_3_Te_14_ are discussed below. Then, considering the close structural relationship of this material with the superconductor Ta_4_Pd_3_Te_16_, a natural question is whether the former is as well a superconductor.

In this paper, we report the observation of SC with *T*_*c*_ = 1.0 K in layered ternary telluride Ta_3_Pd_3_Te_14_ with Q1D PdTe_2_ chains. The bulk SC was identified by the electronic heat capacity data, which shows an obvious anomaly at the transition temperature. The specific-heat jump Δ*C*/(*γ*_*n*_*T*_*c*_) ≈ 1.35 indicates Ta_3_Pd_3_Te_14_ may be a weakly coupled superconductor. In addition, the result of low-temperature thermal conductivity measurements of Ta_3_Pd_3_Te_14_ crystal down to 80 mK suggests a dirty *s*-wave superconducting gap. We summarize our results by discussing the similarities and differences between the closely related superconductors of Ta_3_Pd_3_Te_14_ and Ta_4_Pd_3_Te_16_, and compiled an extended list of their physical properties.

## Results

**Figure 1:**
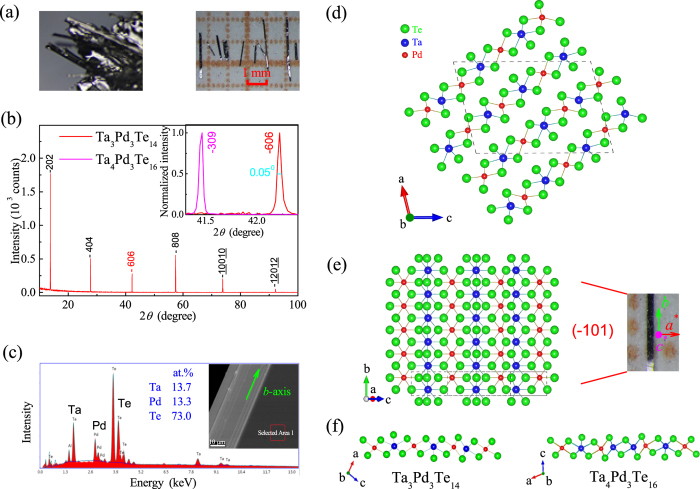
Sample characterization and crystallographic structure of Ta_3_Pd_3_Te_14_. (**a**) Morphology of a batch of the as-grown Ta_3_Pd_3_Te_14_ crystals under an optical microscope (left panel) and photographs of the crystals on a millimeter-grid paper (right panel). (**b**) Single-crystal X-ray diffraction pattern. The inset shows the third inflections in X-ray diffraction pattern for both Ta_3_Pd_3_Te_14_ and Ta_4_Pd_3_Te_16_. (**c**) A typical energy-dispersive X-ray spectrum with electron beams focused on the selected area (marked in the inset) of the crystals. A small amount of the element Al comes from the sample holder. (**d**) Crystal structure of Ta_3_Pd_3_Te_14_ projected along the [010] direction. An individual layer of Ta_3_Pd_3_Te_14_ is shown in the left panel of (**e**). The right panel of (**e**) is a piece of needle-like Ta_3_Pd_3_Te_14_ crystal under an optical microscope, from which the layered morphology can be clearly identified. (**f**) Projection view of one atomic layer of Ta_3_Pd_3_Te_14_ (left) and Ta_4_Pd_3_Te_16_ (right) along the *b* axis.

Single crystals of Ta_3_Pd_3_Te_14_ were grown using a self-flux method, rather than the vapor transport method previously used^21^. Shiny flattened needle-like crystals with a typical size of 2 × 0.15 × 0.1 mm^3^ were harvested, as shown in Fig. 1(a). The X-ray diffraction (XRD) pattern at 298 K by a conventional *θ*-2*θ* scan for the crystals lying on a sample holder is shown in Fig. 1(b), in which we can observe only multiple peaks arising from the diffraction from (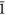 0 1) planes, consistent with the layered crystal structure of Ta_3_Pd_3_Te_14_. Ta_3_Pd_3_Te_14_ crystalizes in space group *P*2_1_/*m* with a monoclinic unit cell of *a* = 14.088(19) Å, *b* = 3.737(3) Å, *c* = 20.560(19) Å, and *β* = 103.73(5)° at 123 K^21^. As seen in Fig. 1(d), the layered slabs compose of successively six different chains of three different types. The three types of chains are Ta-based bicapped trigonal prismatic chains, Pd-based octahedral chains, and Ta-based octahedral chains, respectively. The arrangement of the chains, in such a way that every Pd-based chain has two adjacent Ta-based chains and vice versa, constitute the layered slab as clearly depicted in Fig. 1(e). For simplicity, hereafter we define the a^*^ axis as to be parallel to the[1 0 1] direction and the c^*^ axis as to be perpendicular to the (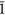 0 1) plane. The interplane spacing at room temperature is determined to be 6.418 Å, and this value is well consistent with the calculated one of 6.397 Å using the above mentioned parameters at 123 K, when taking into account the temperature difference. To compare the obvious difference of the interlayer spacing of Ta_3_Pd_3_Te_14_ and Ta_4_Pd_3_Te_16_, we plot the third reflection together, namely (−606) and (−309) peaks, in the inset of Fig. 1(b), from which one can easily find the interplane spacing of Ta_3_Pd_3_Te_14_ is ~2% smaller than that of Ta_4_Pd_3_Te_16_, and full width at half-maximum is only 0.05°, indicating the high quality of the crystals. The chemical composition determined by an energy-dispersive X-ray spectroscopy (EDS), are collected in a number of crystals, and the average results confirm that composition of the crystals is the stoichiometric Ta_3_Pd_3_Te_14_ within the measurement errors. The SEM image, shown in the upper right corner of Fig. 1(c), has a morphology with stripes along the *b* axis (chain direction), consistent with the preferential crystal growth along the chain direction.

**Figure 2:**
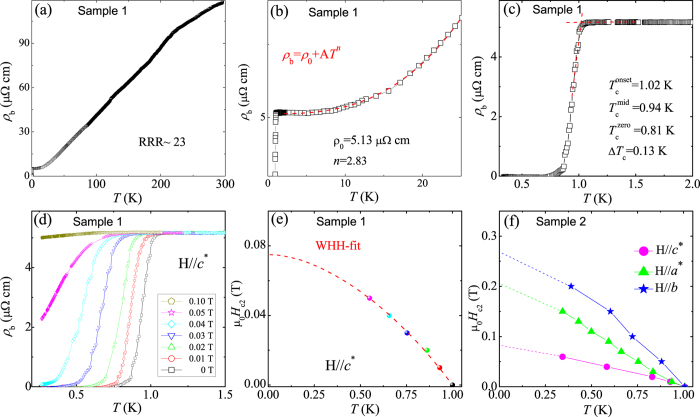
Electrical transport and superconducting phase-diagram. (**a**) Temperature dependence of electronic resistivity of Ta_3_Pd_3_Te_14_ crystal (Sample 1) along the *b* axis. (**b**) shows the power-law fit to *ρ*_*b*_ = *ρ*_0_ + *AT*^*n*^ in the data range of 2 and 25 K. (**c**) zooms into the low-temperature range to clearly show the superconducting transition. (**d**) The low-temperature resistivity in fields H || c^*^ up to 0.1 T, from which the upper critical field (*H*_c2_) is derived. (**e**) The red dashed line represents the Werthamer-Helfand-Hohenberg (WHH) fitting. (**f**) The extracted upper critical field *H*_c2_ of Ta_4_Pd_3_Te_16_ crystal (Sample 2) for different field orientations.

Figure 2(a) shows temperature dependence of electronic resistivity along the *b* axis (*ρ*_*b*_) for the Ta_3_Pd_3_Te_14_ crystal (Sample 1). The larger room temperature resistivity (1.18 *μ*Ω m), than that (0.61 *μ*Ω m) of Ta_4_Pd_3_Te_16_^6^, indicates Ta_3_Pd_3_Te_14_ is less conductive, consistent with the previous reports^21^,^22^. The temperature dependence of resistivity shows a metallic behavior without any obvious anomaly down to *T*_*c*_ = 1.0 K, at which a sharp superconducting transition appears, as clearly depicted in Fig. 2(c). The value of *T*_*c*_ is 3.6 K less than that of Ta_4_Pd_3_Te_16_. The onset, midpoint, and zero-resistance temperatures are 1.02 K, 0.94 K, and 0.81 K, respectively, and the superconducting transition width Δ*T*_*c*_ is 0.13 K. The *ρ*_*b*_(*T*) data between 2 and 25 K can be well fitted by to *ρ*_*b*_ = *ρ*_0_ + *AT*^*n*^, giving a residual resistivity *ρ*_0_ = 5.13 *μ*Ω cm and *n* = 2.83 [Fig. 2(b)]. The value of *n* more than 2 was also observed in Ta_4_Pd_3_Te_16_, which was attributed to the phonon-assisted *s*-*d* interband scattering^18^. The residual resistivity ratio (RRR) is estimated to be RRR = *ρ*_*b*_(300 K)/*ρ*_0_ ~ 23, similar to that of Ta_4_Pd_3_Te_16_ (see Table 1).

Figure 2(d) plots the low-temperature resistivity of Ta_3_Pd_3_Te_14_ crystal (Sample 1) for H || c^*^ up to 0.1 T. Upon increasing the field, the superconducting transition is suppressed to lower temperature. The extracted upper critical fields *H*_c2_(*T*) for H || c^*^, determined by using 90% criterion, *i*.*e*., the field at which *ρ*_*b*_ reaches 90% of the normal state resistivity, are shown in Fig. 2(e). We applied the isotropic one-band Werthamer-Helfand-Hohenberg (WHH) formalism to roughly estimate *H*_c2_^23^. As can be seen in Fig. 2(e), *H*_c2_ for H || c^*^ is estimated to be 0.075 T at zero temperature with the derived Maki parameter *α* = 1.7 and spin-orbit coupling parameter *λ*_so_ = 1.2. However, by employing the orbital limiting field 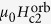
(0) = −0.69*μ*_0_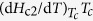 = 0.1 T in WHH model and the BCS Pauli-limiting field 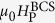 = 1.84*T*_*c*_ = 1.84 T, the Maki parameter *α* = 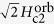
(0)/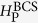 is calculated to be 0.077. This inconsistence between the calculated value of *α* and the fitted one may originate from the anisotropic effect in Ta_3_Pd_3_Te_14_. The extracted *H*_c2_ with fields applied along a^*^, *b*, and c^*^ directions for Sample 2 are shown in Fig. 2(f), and the resistivity data of Sample 2 are not shown here. By roughly linear extrapolations, the anisotropic *H*_c2_ at zero temperature are estimated to be 0.21, 0.27 and 0.086 T for the a^*^, *b* and c^*^ directions. Using the Ginzburg-Landau formula, the superconducting coherence length *ξ* are calculated to be 545, 703 and 223 Å for a^*^, *b* and c^*^ directions, respectively, which are much larger than those of its analog Ta_4_Pd_3_Te_16_^17^. The SC in Ta_3_Pd_3_Te_14_ is anisotropic but as well three-dimensional in nature, similar to other superconductors with Q1D characteristics, *e*.*g*., Ta_4_Pd_3_Te_16_^17^, Nb_3_Pd_0.7_Se_7_^24^, and Nb_2_Pd_*x*_Se_5_^25^, since the interchain coherence length 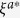 and 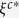 are much larger than the distance between two arbitrarily adjacent chains.

**Figure 3:**
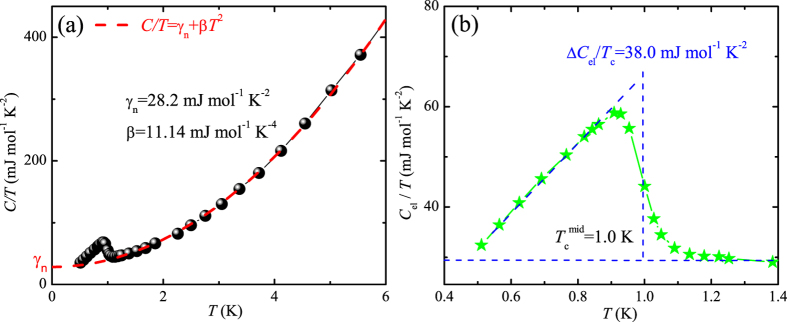
Temperature dependence of specific heat. (**a**) *C*/*T* vs *T*, in which the red dashed line represents the fit with the formula *C*/*T* = *γ*_*n*_ + *βT*^2^ for the normal-state data from 1.2 to 6.5 K. (**b**) The electronic specific heat divided by temperature *C*_el_/*T* in the superconducting state, where *C*_el_ = *C* − *βT *^3^.

The low-temperature specific heat data of Ta_3_Pd_3_Te_14_ crystals, plotted as *C*/*T* vs *T*, are shown in Fig. 3(a). We fit the normal-state data from 1.2 to 6.5 K, employing the usual formula *C*/*T* = *γ*_*n*_ + *βT*^2^, which is represented as the red dashed line. The fitting yields an electronic heat capacity coefficient *γ*_*n*_ = 28.2 ± 0.9 mJ mol^−1^ K^−2^, and a phononic coefficient *β* = 11.14 ± 0.04 mJ mol^−1^ K^−4^. The calculated Debye temperature Θ_D_ = 151.6 K is close to the value of Ta_4_Pd_3_Te_16_, consistent with the fact that the structures of two tellurides are closely related. However, the value of extracted coefficient *γ*_*n*_ is nearly 40% smaller than that of Ta_4_Pd_3_Te_16_^17^. Using the relation *N*(*E*_F_) = 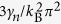 for noninteracting electron systems, where *k*_B_ is the Boltzmann constant, we estimated the density of states at the Fermi level *N*(*E*_F_) to be about 11.9 ± 0.8 eV^−1^ fu^−1^, which is 3.5 times that of the bare density of states *N*_bs_(*E*_F_), obtained from the previous band-structure calculations^20^. Therefore, the larger renormalization factor [*N*(*E*_F_)/*N*_bs_(*E*_F_) = 1 + *λ*], than that for Ta_4_Pd_3_Te_16_, suggests much stronger electron-electron correlations in Ta_3_Pd_3_Te_14_, although the recent band-structure calculations are concluded with a higher *N*_bs_(*E*_F_) = 9.6 eV^−1^ fu^−1^ for Ta_4_Pd_3_Te_16_, thus resulting in a much lower renormalization factor^19^. To extract the electron-nonphonon coupling strength *λ*_nph_ in *λ*, we estimate the electron-phonon coupling constant *λ*_ph_ by employing the McMillan formula^26^, *λ*_ph_ = [1.04 + μ^*^ln(Θ_D_/1.45*T*_*c*_)]/[(1 − 0.62μ^*^)ln(Θ_D_/1.45*T*_*c*_) − 1.04], where the Coulombic repulsion parameter μ^*^ is empirically set to be 0.13. The estimated value of *λ*_ph_ is 0.51, a little bit smaller than that of the superconductor Ta_4_Pd_3_Te_16_^6^. However, the resultant constant *λ*_nph_ = *λ* − *λ*_ph_ = 1.99 is much larger than that of Ta_4_Pd_3_Te_16_, possibly indicating much larger electron correlations in the former compound.

## Discussion

We discuss the electronic heat capacity *C*_el_ of Ta_3_Pd_3_Te_14_ crystals in low-temperature range, obtained by *C*_el_ = *C* − *βT *^3^. As can be seen in Fig. 3(b), a characteristic superconducting jump (Δ*C*_el_) shows up around ~1 K, confirming the bulk SC. The Δ*C*_el_/*T*_*c*_ is estimated to be 38.0 mJ mol^−1^ K^−2^ and the midpoint temperature of the thermodynamic transition is 1.0 K, consistent with the superconducting transition in low-temperature resistivity. The dimensionless specific-heat jump can be calculated to be 1.35, smaller than the theoretical value (1.43) of the well-known BCS theory, indicating Ta_3_Pd_3_Te_14_ may be a weakly coupled superconductor. Unfortunately, due to the insufficient data points, we are unable to fit Δ*C*_el_(*T*) with standard gap functions to give valuable information about the gap symmetry.

**Figure 4:**
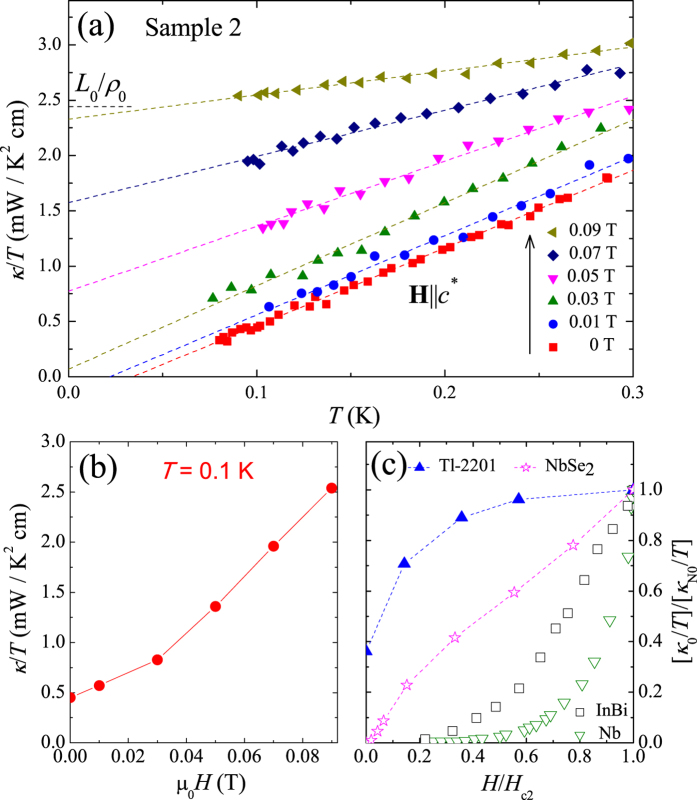
Low-temperature thermal conductivity data. (**a**) Low-temperature thermal conductivity of Ta_3_Pd_3_Te_14_ crystal (Sample 2) in zero and magnetic fields applied along c^*^ direction. The dashed lines are fits to the formula *κ*/*T* = *κ*_0_/*T* + *bT*. The black dashed line is the normal-state Wiedemann-Franz law expectation *L*_0_/*ρ*_0_. (**b**) The field dependence of *κ*/*T* at 0.1 K. (**c**) Normalized residual linear term *κ*_0_/*T* as a function of normalized field *H*/*H*_*c*2_ for the clean *s*-wave superconductor Nb^36^, the dirty *s*-wave superconducting alloy InBi^32^, the multi-band *s*-wave superconductor NbSe_2_^37^, and an overdoped *d*-wave cuprate superconductor Tl-2201^38^.

To shed light on the superconducting gap structure, we measured the thermal conductivity of Ta_3_Pd_3_Te_14_ single crystal (Sample 2) in zero and magnetic fields (along c^*^ direction), the results of which are plotted as *κ*/*T* vs *T* in Fig. 4(a). Since all the curves presented in Fig. 4(a) are roughly linear as previously reported in Ta_4_Pd_3_Te_16_^18^ and some iron-based superconductors^27^,^28^, we fit all the curves to *κ*/*T* = *a* + *bT*^*α*−1^ by fixing *α* to 2. The two terms *aT* and *bT*^*α*^ represent contributions from electrons and phonons, respectively^29^,^30^. From the curve in magnetic field *H* = 0.09 T, which is close to the critical field *H*_*c*2_(0) for ***H ***|| c^*^, one can see that the obtained *κ*_0_/*T* roughly meets the normal-state Wiedemann-Franz law expectation *κ*_N0_/*T* = *L*_0_/*ρ*_0_ = 2.44 mW K^−2^ cm^−1^. Here, *L*_0_ is the Lorenz number 2.45 × 10^−8^ W Ω K^−2^ and *ρ*_0_ = 10.04 *μ*Ω cm is the residual resistivity of Sample 2. The verification of the Wiedemann-Franz law in the normal state demonstrates that our thermal conductivity measurements are reliable. For the curves in *H* = 0 and 0.01 T, however, the linear fittings give two negative values, *κ*_0_/*T* = −0.24 and −0.16 mW K^−2^ cm^−1^, respectively. These negative *κ*_0_/*T* have no physical meaning, just because the temperature of our measurement is not low enough, comparing to the *T*_*c*_. Down to lower temperature, the curve in zero field should deviate from the linear behavior.

Since we can not extrapolate *κ*_0_/*T* at low field, we plot the field dependence of *κ*/*T* at *T* = 0.1 K, well below *T*_*c*_, in Fig. 4(b) to get more information about the superconducting gap structure of Ta_3_Pd_3_Te_14_^31^. One can see that the increase of *κ*/*T* at low field is rather slow, and the curve is similar to that of dirty *s*-wave superconduting alloy InBi^32^, which is shown in Fig. 4(c). By using the estimated value of the coherence length *ξ* along *b* direction, the formula *ξ* = 0.18*ħυ*_*F*_/*k*_*B*_*T*_*c*_ gives the Fermi velocity *υ*_*F*_ = 5.11 × 10^4^ m s^−1^ 33. Then, according to the relationship *κ*_N0_/*T* = *γ*_*n*_*υ*_*F*_*l*_*e*_/3, the electron mean free path is estimated to be *l*_*e*_ = 322 Å, which is much smaller than the *b*-direction *ξ*. This result indicates Ta_3_Pd_3_Te_14_ is indeed in the dirty limit. Therefore, it is concluded that Ta_3_Pd_3_Te_14_ is very likely a dirty *s*-wave superconductor.

It is instructive to compare the physical properties of the two structural closely related compounds of Ta_3_Pd_3_Te_14_ and Ta_4_Pd_3_Te_16_, which we summarize in Table 1. Both of the two tellurides show Q1D characteristic with Q1D PdTe_2_ chains. The larger RRR could account for the much sharper superconducting transition for Ta_3_Pd_3_Te_14_. Although *T*_*c*_ of Ta_3_Pd_3_Te_14_ is 3.6 K less than that of Ta_4_Pd_3_Te_16_, the former compound show much stronger electron correlations, verified by the larger renormalization factor and larger electron-nonphonon coupling strength *λ*_nph_. The small values of both Δ*C*/(*γ*_*n*_*T*_*c*_) and *λ*_ph_ indicate Ta_3_Pd_3_Te_14_ is a weakly coupled superconductor. In addition, if assuming the Drude model, in which the electron-electron interactions are neglected, is applicable, a superconductor is expected to have a lower *T*_*c*_ for a lower value of Sommerfield coefficient *γ*_*n*_, which in this case signifies the lower density of states at Fermi level. This simple conclusion is compatible with the general trend for the above two superconductors. Therefore, the lower *T*_*c*_ in the title compound, may be attributed to the lower density of states at Fermi level. In this sense, by tuning the Fermi level of Ta_3_Pd_3_Te_14_ or Ta_4_Pd_3_Te_16_ by the way of doping or proper intercalations, the value of *T*_*c*_ may be enhanced. By the way, in recently discovered PdTe or PdS chains based superconductors, there have been several pieces of work that show the evidences of two-gap SC^17^,^34^,^35^. However, our results presented above indicate the new superconductor Ta_3_Pd_3_Te_14_ with PdTe_2_ chains is very likely a fully gapped *s*-wave one. We have previously reported that Ta_4_Pd_3_Te_16_ is possibly a two-gap superconductor with a gap symmetry of *s* + *d* waves^17^. Thus, if assuming the reduced part of electronic states at the Fermi level in Ta_3_Pd_3_Te_14_, compared to that in Ta_4_Pd_3_Te_16_, is primarily due to the reduced contribution from the Pd *d* states, it would be reasonable to see only a *s*-wave gap left in the former compound.

## Methods

Powders of the elements Ta (99.97%), Pd (99.995%) and Te (99.99%) with a ratio of Ta : Pd : Te = 2 : 3 : 10 were thoroughly mixed together, loaded, and sealed into an evacuated quartz ampule. The sample-loaded quartz ampoule is then heated to 1223 K, held for 24 h, and cooled to 723 K at a rate of 5 K/h, followed by furnace cooling to room temperature. The above procedures are similar to that in growing Ta_4_Pd_3_Te_16_ crystals^6^. The chemical composition is checked by an EDS with an AMETEK EDAX (Model Octane Plus) spectrometer, equipped in a field-emitting scanning electron microscope (SEM, Hitachi S-4800). It is worthy to mention that, although one may speculate the as-grown Ta_3_Pd_3_Te_14_ crystals should be mixed by the Ta_4_Pd_3_Te_16_ crystals in the same batch, our results do not support this speculation. The reasons are as follows: Using this method and atomic ratio of Ta : Pd : Te = 2 : 3 : 10 to grow Ta_3_Pd_3_Te_14_ crystals, Ta_4_Pd_3_Te_16_ crystals were occasionally harvested. However, our results show that the two kinds of crystals are never mixed with each other in the same batch. This conclusion is drawn by the fact that once the crystals, randomly picked up from the final product in one batch, are checked by both XRD and EDS to be Ta_3_Pd_3_Te_14_, not a single piece of Ta_4_Pd_3_Te_16_ crystals can be identified in the same batch, and vise versa.

The magnetoresistance (MR) measurements were carried out in a ^3^He cryostat down to 0.27 K by a stand four-probe technique with current applied along the *b* axis. The specific heat for a bundle of shiny needle-like crystals with a total mass *m* = 1.30(2) mg was measured by a long relaxation method utilizing a commercial ^3^He microcalorimeter (Quantum Design PPMS-9). The thermal conductivity was measured in a commercial dilution refrigerator, using a standard four-wire steady-state method with two RuO_2_ chip thermometers, calibrated *in situ* against a reference RuO_2_ thermometer. The chemical composition of the crystals employed for above measurements have also been checked to be Ta_3_Pd_3_Te_14_ by both XRD and EDS.

## Additional Information

**How to cite this article**: Jiao, W.-H. *et al.* Superconductivity in Ta_3_Pd_3_Te_14_ with quasi-one-dimensional PdTe_2_ chains. *Sci. Rep.* **6**, 21628; doi: 10.1038/srep21628 (2016).

## Figures and Tables

**Table 1:** Comparison of some physical parameters of the superconductors Ta_3_Pd_3_Te_14_ (present work and^20^) and Ta_4_Pd_3_Te_16_ 6,17,19,20.

Space group	Ta_3_Pd_3_Te_14_	Ta_4_Pd_3_Te_16_
	*P*2_1_/*m*	*I*2/*m*
Physical parameters		
RRR	23	26
*T*_*c*_ (K)	1.0	4.6
Δ*T*_*c*_ (K)	0.13	0.76
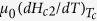 (T/K) (***H*** ***||*** c^*^)	−0.14	−0.44
*μ*_0_*H*_c2_ (T) (***H*** ***||*** c^*^)	0.075	3.3
*γ*_*n*_ (mJ mol^−1^ K^−2^)	28.2	46.1
*θ*_*D*_ (K)	151.6	148.8
*λ*_ph_	0.51	0.77
*λ*_nph_	1.99	1.53^6^, 0.12^19^
*N*_bs_(*E*_F_) (eV^−1^ fu^−1^)	3.4^20^	9.6^19^, 5.5^20^
Δ*C*/(*γ*_*n*_*T*_*c*_)	1.35	1.40

RRR, *T*_*c*_, Δ*T*_*c*_, *H*_*c*2_, *γ*_*n*_, *θ*_*D*_, *λ*_ph_, *λ*_nph_, *N*_bs_(*E*_F_), and Δ*C*/(*γ*_*n*_*T*_*c*_) denote the residual resistivity ratio, superconducting transition temperature, transition width, upper critical field, electronic specific-heat coefficient, Debye temperature, electron-phonon coupling constant, electron-nonphonon coupling constant, density of states at Fermi level, and dimensionless specific-heat jump, respectively.

## References

[b1] Bednorz J. G. & Müller K. A. Possible high T_*c*_ superconductivity in the BaLaCuO system. Z. Phys. B 64, 189 (1986).

[b2] Maeno Y. *et al.* Superconductivity in a layered perovskite without copper. Nature 372, 532 (1994).

[b3] Kamihara Y., Watanabe T., Hirano M. & Hosono H. Iron-based layered superconductor La[O_1−*x*_F_*x*_]FeAs (*x* = 0.05–0.12) with T*c* = 26 K. J. Am. Chem. Soc. 130, 3296 (2008). 18293989 10.1021/ja800073m

[b4] Mizuguchi Y. *et al.* BiS_2_-based layered superconductor Bi_4_O_4_S_3_. Phys. Rev. B 86, 220510(R) (2012).

[b5] Zhang Q. *et al.* Superconductivity with extremely large upper critical fields in Nb_2_Pd_0.81_S_5_. Sci. Rep. 3, 1446 (2013). 23486091 10.1038/srep01446 PMC3595695

[b6] Jiao W. H. *et al.* Superconductivity in a layered Ta_4_Pd_3_Te_16_ with PdTe_2_ chains. J. Am. Chem. Soc. 136, 1284 (2014). 24428401 10.1021/ja412094n

[b7] Goyal R., Tiwari B., Jha R. & Awana V. P. S. Superconductivity at 4.4 K in PdTe_2_-chains of a Ta based compound. J. Supercond. Nov. Magn. 28, 1195 (2015).

[b8] Bao J. K. *et al.* Superconductivity in quasi-one-dimensional K_2_Cr_3_As_3_ with significant electron correlations. Phys. Rev. X 5, 011013 (2015).

[b9] Stewart G. R. Superconductivity in iron compounds. Rev. Mod. Phys. 83, 1589 (2011).

[b10] Yasuhiro T., Youichi Y. & Masao O. Superconductivity in Na_*x*_CoO_2_·*y*H_2_O due to charge fluctuation. J. Phys. Soc. Jpn. 73, 319 (2004).

[b11] Lee P. A., Nagaosa N. & Wen X. G. Doping a mott insulator: Physics of high-temperature superconductivity. Rev. Mod. Phys. 88, 033620 (2013).

[b12] Lefebvre S. *et al.* Mott transition, antiferromagnetism, and unconventional superconductivity in layered organic superconductors. Phys. Rev. Lett. 85, 5420 (2000). 11136011 10.1103/PhysRevLett.85.5420

[b13] Mitchell K. & Ibers J. A. Rare-earth transition-metal chalcogenides. Chem. Rev. 102, 1929 (2002). 12059258 10.1021/cr010319h

[b14] Wilson J. A., Di Salvo F. J. & Mahajan S. Charge-density waves and superlattices in the metallic layered transition metal dichalcogenides. Adv. Phys. 24, 117 (1975).

[b15] Tritt T. M. Holey and unholey semiconductors. Science 283, 804 (1999).

[b16] Du Z. Y. *et al.* Anisotropic superconducting gap and elongated vortices with caroli-de gennes-matricon states in the new superconductor Ta_4_Pd_3_Te_16_. Sci. Rep. 5, 9408 (2015). 25797138 10.1038/srep09408 PMC4369749

[b17] Jiao W. H. *et al.* Multiband superconductivity in Ta_4_Pd_3_Te_16_ with anisotropic gap structure. J. Phys.: Cond. Matt. 27, 325701 (2015). 10.1088/0953-8984/27/32/325701 26214563

[b18] Pan J. *et al.* Nodal superconductivity and superconducting dome in the layered superconductor Ta_4_Pd_3_Te_16_. Phys. Rev. B 92, 180505(R) (2015).

[b19] Singh D. J. Multiband superconductivity of Ta_4_Pd_3_Te_16_ from Te *p* states. Phys. Rev. B 90, 144501 (2014).

[b20] Alemany P., Jobic S., Brec R. & Canadell E. Oxidation states, transport properties, and Te···Te short contacts in the ternary transition metal tellurides Ta_3_Pd_3_Te_14_ and Ta_4_Pd_3_Te_16_. Inorg. Chem. 36, 5050 (1997).

[b21] Liimatta E. W. & Ibers J. A. Synthesis, structures, and conductivities of the new layered compounds Ta_3_Pd_3_Te_14_ and TaNiTe_5_. J. Solid State Chem. 78, 7 (1989).

[b22] Mar A. & Ibers J. A. Synthesis, crystal structure and electrical conductivity of a new layered ternary telluride Ta_4_Pd_3_Te_16_. J. Chem. Soc., Dalton Trans. S, 639 (1991).

[b23] Werthamer N. R., Helfand E. & Hohenberg P. C. Temperature and purity dependence of the superconducting critical field, *H*_*c*2_. III. Electron spin and spin-orbit effects. Phys. Rev. 147, 295 (1966).

[b24] Zhang Q. R. *et al.* Anomalous metallic state and anisotropic multiband superconductivity in Nb_3_Pd_0.7_Se_7_. Phys. Rev. B 88, 024508 (2013).

[b25] Khim S. *et al.* Enhanced upper critical fields in a new quasi-one-dimensional superconductor Nb_2_Pd_*x*_Se_5_. New J. Phys. 15, 123031 (2013).

[b26] McMillan W. L. Transition temperature of strong-coupled superconductors. Phys. Rev. 167, 331 (1968).

[b27] Dong J. K. *et al.* Quantum criticality and nodal superconductivity in the FeAs-based superconductor KFe_2_As_2_. Phys. Rev. Lett. 104, 087005 (2010). 20366962 10.1103/PhysRevLett.104.087005

[b28] Qiu X. *et al.* Robust nodal superconductivity induced by isovalent doping in Ba(Fe_1−*x*_Ru_*x*_)_2_As_2_ and BaFe_2_(As_1−*x*_P_*x*_)_2_. Phys. Rev. X 2, 011010 (2012).

[b29] Li S. Y. *et al.* Low-temperature phonon thermal conductivity of single-crystalline Nd_2_CuO_4_: Effects of sample size and surface roughness. Phys. Rev. B 77, 134501 (2008).

[b30] Sutherland M. *et al.* Thermal conductivity across the phase diagram of cuprates: Low-energy quasiparticles and doping dependence of the superconducting gap. Phys. Rev. B 67, 174520 (2003).

[b31] Shakeripour H., Petrovic C. & Taillefer L. Heat transport as a probe of superconducting gap structure. New J. Phys. 11, 055065 (2009).

[b32] Willis J. O. & Ginsberg D. M. Thermal-conductivity of superconducting alloy-films in a perpendicular magnetic-field. Phys. Rev. B 14, 1916 (1976).

[b33] See, for example, Tinkham M. Introduction to superconductivity (McGraw-Hill, New York, 1975).

[b34] Goyal R., Tiwari B., Jha R. & Awana V. P. S. Specific heat of robust Nb_2_PdS_5_ superconductor. J. Supercond. Nov. Magn. 28, 1427 (2015).

[b35] Park E. *et al.* Spectroscopic evidence for two-gap superconductivity in the quasi-one dimensional chalcogenide Nb_2_Pd_0.81_S_5_. Preprinted at http://arxiv.org/abs/1505.01258 (2015).

[b36] Lowell J. & Sousa J. B. Mixed-state thermal conductivity of type II superconductors. J. Low Temp. Phys. 3, 65 (1970).

[b37] Boaknin E. *et al.* Heat conduction in the vortex state of NbSe_2_: Evidence for multiband superconductivity. Phys. Rev. Lett. 90, 117003 (2003). 12688957 10.1103/PhysRevLett.90.117003

[b38] Proust C., Boaknin E., Hill R. W., Taillefer L. & Mackenzie A. P. Heat transport in a strongly overdoped cuprate: Fermi liquid and a pure *d*-wave BCS superconductor. Phys. Rev. Lett. 89, 147003 (2002). 12366068 10.1103/PhysRevLett.89.147003

